# GMG-LDefmamba-YOLO: An Improved YOLOv11 Algorithm Based on Gear-Shaped Convolution and a Linear-Deformable Mamba Model for Small Object Detection in UAV Images

**DOI:** 10.3390/s25226856

**Published:** 2025-11-10

**Authors:** Yiming Yang, Lingyu Yan, Jing Wang, Jinhang Liu, Xing Tang

**Affiliations:** 1 School of Computer Science, Hubei University of Technology, Wuhan 430068, China; yangyiming@hbut.edu.cn (Y.Y.); wangjing@hbut.edu.cn (J.W.); liujinhang@hbut.edu.cn (J.L.); 2 Key Laboratory of Green Intelligent Computing Network in Hubei Province, Wuhan 430068, China; 3 School of Computer Science and Artificial Intelligence, Wuhan University of Technology, Wuhan 430070, China; tangxing@whut.edu.cn

**Keywords:** UAV remote sensing image, small object detection, YOLOv11, gear shape convolution, linear deformable mamba

## Abstract

Object detection plays a crucial role in remote sensing and UAV image technology, but it faces the challenge of speed and accuracy in multi-scale dense small target mission detection scenarios and is susceptible to noise interference, such as weather conditions, lighting changes, and occluded backgrounds in complex backgrounds. In recent years, Mamba-based methods have become hot in the field of object detection, showing great potential in capturing remote dependencies with linear complexity but lacking deep customization of remote sensing targets. Based on this, we propose GMG-LDefmamba-YOLO, which contains two core modules: the Gaussian mask gear convolution module forms a gear-shaped receptive field through improved convolutional splicing to enhance the extraction of small target features and combines the Gaussian mask mechanism to dynamically modulate the feature weights to suppress complex background interference. The linear deformable Mamba module integrates linear deformable sampling, a spatial state dual model, and residual gating MLP components, integrating the advantages of flexible capture of local features and efficient modeling of global dependence, dynamically adapting to target scale changes and spatial distribution, and reducing computational costs. Experiments on DOTA-v1.0, VEDAI, and USOD datasets show that the mAP50 of the model reaches 70.91%, 77.94%, and 90.28%, respectively, which is better than the baseline and mainstream methods, and maintains the lightweight characteristics, providing efficient technical support for remote sensing monitoring, UAV inspection, and other fields.

## 1. Introduction

**Figure 1:**
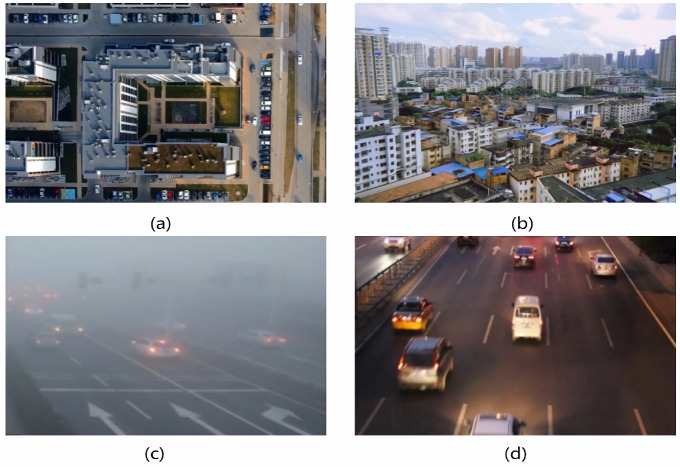
Challenges faced by remote sensing target detection. (**a**) Dense small targets, (**b**) complex backgrounds, (**c**) blurred features, and (**d**) dim lighting conditions.

In recent years, remote sensing target detection technology (RSIs) [1] and unmanned aerial vehicle detection technology (UAV) [2] have been used in agricultural monitoring [3] and environmental monitoring [4], infrastructure inspection [5], and other fields, providing efficient data support for the acquisition and analysis of wide-area geographic information. However, target detection in remote sensing and UAV images still faces many challenges as shown in Figure 1: on the one hand, remote sensing targets are usually dense and have a very low proportion of pixels (some are even less than 16 × 16 pixels), the feature information is sparse and highly dependent on the central feature for positioning, and its feature representation is vague and extremely susceptible to interference from complex backgrounds [6]; On the other hand, the scale of remote sensing targets changes drastically, and the target morphology at different distances varies significantly, coupled with factors such as changes in lighting conditions and target occlusion [7], which further exacerbates the difficulty of accurate detection. Especially the balance between the detection accuracy of small targets and the efficiency of the algorithm needs to be solved urgently.

In the field of object detection, deep learning-based algorithms have become mainstream and are mainly divided into two-stage detection algorithms and single-stage detection algorithms according to their workflow. Two-stage detection algorithms [8], such as the R-CNN series [9], first generate regional suggestions and then extract features and classify these regions. Although this method achieves higher detection accuracy, it is computationally expensive and slow, making it unsuitable for scenarios that require rapid response. The Faster R-CNN [10] algorithm significantly improves detection speed by introducing a region suggestion network, but it still requires classification and bounding box regression for each candidate region, which limits its performance in real-time applications. In contrast, SSDs [11] pioneered single-stage detection, followed by methods such as RetinaNet [12] and YOLO [13]. In addition to these two detection algorithms, there is a class of visual transformers, including DETR [14] and Swin Transformer [15], which process images by dividing them into contiguous chunks and applying self-attention mechanisms for object detection. While vision converters can simplify the overall architecture, their high computational requirements make them unsuitable for deployment on resource-constrained drone equipment. In contrast, single-stage detection algorithms directly predict object classification and bounding boxes on the entire image without generating area suggestions, resulting in faster detection speeds, and are suitable for real-time applications and UAV platform integration. Notably, the YOLO series has garnered attention for its fast inspection speed and balanced performance, maintaining inspection accuracy while ensuring speed. However, single-level algorithms generally lag slightly behind two-level algorithms in terms of accuracy, especially in small object detection.

In recent years, more work has begun to rethink how to design improved CNNs to achieve higher accuracy and faster speeds. More and more studies have begun to use hybrid architectures to improve models and reduce complexity, such as MobileVit [16], EdgeVit [17], and EfficientFormer [18]. The state space model (SSM) Mamba [19], equipped with a selective scanning mechanism, has shown superior performance in long-distance interaction and linear computational complexity. These advantages enable it to effectively address the computational inefficiencies of transformers in long-sequence spatial modeling. The model LS-MambaNet is based on the improved scanning mechanism of Mamba [20] and the hybrid architecture Mamba-YOLO [21]. Excellent progress has been made in this regard. At the same time, other innovations for convolution methods are also present, such as the improved splicing method of convolution, pinwheel-shaped convolution [22], and deformable convolution DCNv4 [23]. Outstanding results have been achieved in the field of object detection. However, these methods still have deficiencies in adapting to the characteristics of small targets in the field of remote sensing, and their computing power has obvious limitations in terms of detection speed and adaptability to edge devices such as drones. However, although the improved convolutional splicing method strengthens the directional sensitivity, it lacks edge feature processing ability and is susceptible to complex background interference from remote sensing targets. Although traditional deformable convolution can adapt to the multi-scale morphological changes in remote sensing targets, the computational cost is too high due to the parameter growth mode, and performance bottlenecks are prone to occur on edge devices with limited computing power. Therefore, it is crucial to explore the combination of advanced methods and the YOLO architecture.

To solve the above challenges, this paper proposes a target detection method based on the YOLOv11 framework, GMG-LDefmamba-YOLO, specifically designed for remote sensing small object detection. The main contributions of this study are as follows:

1. The Gaussian mask gear convolutional GMGblock module is proposed, which extracts directional sensitivity features through the mirror-symmetrical eight-way padding convolutional kernel branch, splices it to form a symmetrical receptive field of gear shape, and combines the dynamic modulation of the Gaussian mask mechanism to strengthen the central feature extraction ability of small targets and effectively suppress complex background interference.
2. The linear deformable Mamba block LDefMambablock module is designed, integrating the linear deformable sampling LDblock, the spatial state dual model SS2D, and the residual gating MLP component, integrating the flexible local feature capture capability of linear deformable convolution and the efficient global dependency modeling advantages of the Mamba architecture, dynamically adapting to the spatial distribution and scale changes in the target, and the gating mechanism optimizes the computing efficiency while ensuring accuracy, and adapts to the computing power constraints of edge devices.
3. The proposed GMG-LDefmamba-YOLO architecture achieves the most advanced detection performance in the field of remote sensing small targets. Excellent detection accuracy and inference efficiency were demonstrated on DOTA-v1.0, VEDAI, and USOD datasets.

Notably, our work differs from existing Mamba-YOLO in two critical ways: (1) We adopt YOLOv11 as the baseline (instead of YOLOv8) and explore SSM’s compatibility with its newly added modules (SPPF, C2PSA) and core C3k2 module, addressing the limitation that most YOLO improvements focus on older versions (v5/v8); (2) We customize the method for remote sensing small target detection, while Mamba-YOLO targets general object detection. These differences lay the foundation for our methodological innovations and further verify the compatibility of Mamba with the YOLO architecture. At the same time, we compare the newer YOLO versions, including YOLOv12 and YOLOv13, in the experiment, and demonstrate the SOTA performance of YOLOv11 through experimental results. The source code will be available at https://github.com/acaneyoru/GMG-LDefmamba-YOLO (accessed on 2 July 2025).

## 2. Related Works

### 2.1. YOLOv11

**Figure 2:**
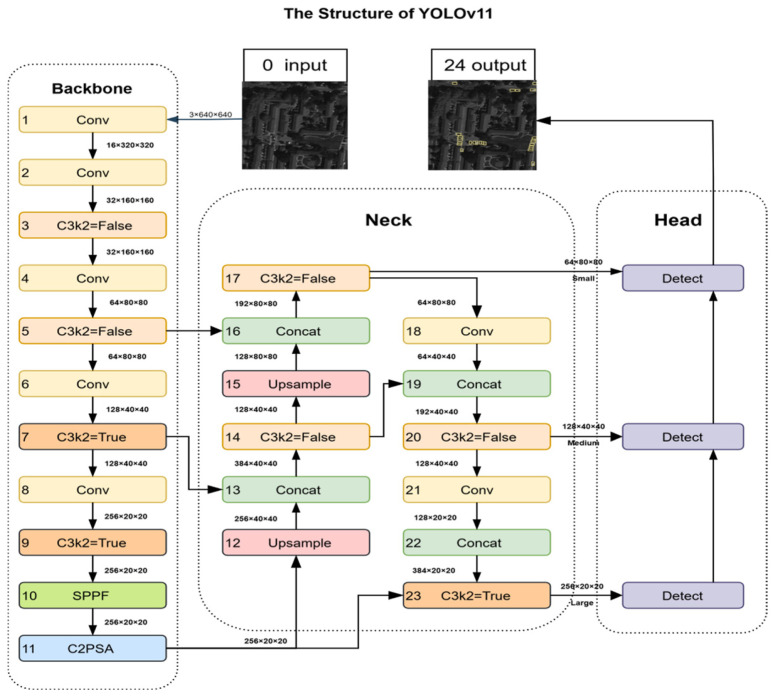
Structure of YOLOv11.

As shown in Figure 2, the YOLOv11 [24] architecture consists of three key components: the backbone, neck, and head. The YOLOv11 backbone network retains the modular hierarchical design but replaces the original C2f module with a more efficient C3k2 unit and adds a convolutional module C2PSA with spatial attention [25] to enhance the detection of small and occluded targets. At the same time, YOLOv11’s spatial pyramid fast pooling SPPF is retained as an upgraded version of traditional SPP for sequential pooling to achieve excellent feature representation [26]. The neck of YOLOv11 adopts a path aggregation network PANet structure [27] to enable cross-scale feature interaction through upsampling and concatenation stitching operations. The neck integrates the different-level features output by the trunk, which not only retains the spatial details in the low-level features but also integrates the semantic information of the high-level features, providing rich feature support for small object detection. The detection head part continues the efficiency of the YOLO series of single-stage detection, using multi-scale detection branches (corresponding to small, medium, and large targets) combined with box_loss, cls_loss, and dfl_loss for joint optimization [28]. Through the collaborative design of the backbone, neck, and head, YOLOv11 achieves a balance between detection accuracy and inference efficiency while maintaining lightweight characteristics.

### 2.2. State Space Models

In recent years, structural state space sequence models (SSMs) such as Mamba [29] have become powerful methods for long sequence modeling. The model has the advantage of input-scale linear complexity and can efficiently model global information. SSM reduces computational complexity from quadratic to linear by compressing hidden states that allow each element in a one-dimensional sequence (e.g., a text sequence) to interact with a previously scanned sample compared to traditional self-injection mechanisms. While SSM was originally designed for natural language processing (NLP) tasks, it has also shown great potential in the field of computer vision. Vision Mamba [30] proposed a pure visual backbone model based on selective SSM, marking the first introduction of Mamba into the visual field. VMamba [31] introduced a cross-scan module that enables the model to selectively scan 2D images, enhance vision processing capabilities, and demonstrate superiority in image classification tasks. LocalMamba [32] focuses on window scanning strategies for visuospatial models, optimizing visual information to capture local dependencies, and introducing dynamic scanning methods to search for optimal options for different layers. Although the above-mentioned Mamba-based model shows excellent performance in vision tasks, the scanning strategy in SSM introduces additional computational costs and functional redundancy, which becomes a performance bottleneck for remote sensing target recognition. In addition, although the Mamba method strengthens the connection between the spatial distribution of targets, its ability in local feature extraction is limited, especially in the feature extraction of small targets. At the same time, as an end-to-end detector, we hope that the improved Mamba model based on the YOLO architecture does not need to be pre-trained on large-scale datasets but is directly trained on the target dataset and put into use.

### 2.3. Deformable Convolution

Traditional convolutional networks with fixed receptive fields are difficult to adapt to the dynamic characteristics of aerial scenes [33], so lightweight and adaptive feature extraction methods are needed to balance small target sensitivity and computational efficiency. Dai et al. [34] developed a deformable convolutional network (DCN) using a dynamically adjustable offset mechanism, which significantly enhanced the spatial adaptability of convolutional kernels. However, the multi-layer deformable structure proliferates the model parameters, exacerbating the consumption of computational resources during training. Du et al. [35] proposed a sparse convolution scheme that applies channel pruning optimization to the detection head, which reduces computational complexity while maintaining detection accuracy. However, this approach compromises feature discernibility in complex contexts. Wang et al. [36] constructed an elastic receptive field model using deformable convolution as a basic operator, breaking the traditional geometric constraints. However, a mechanism linking receptive fields to the spatial distribution of small targets has not been established. Qi et al. [37] developed DSCNet with a serpentine convolutional structure, which improved the segmentation accuracy of the network. However, the topological feature extraction mechanism is susceptible to irregular shape interference in general object detection scenarios, and the stability of dynamic kernel adjustment needs to be optimized. The latest linear deformable method, LDConv [38], is considered to be very effective in improving the field of small target feature extraction, Wu et al. [39]. Combining this method with YOLO architecture, the AAPW-YOLO network is proposed for remote sensing target detection. However, this method does not perform well in dense object detection.

## 3. Method

### 3.1. Overview of GMG-LDefmamba-YOLO

**Figure 3:**
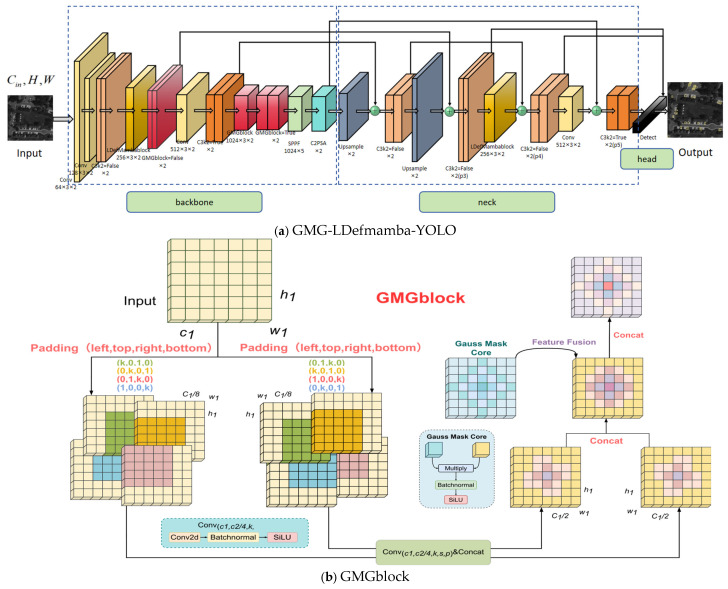

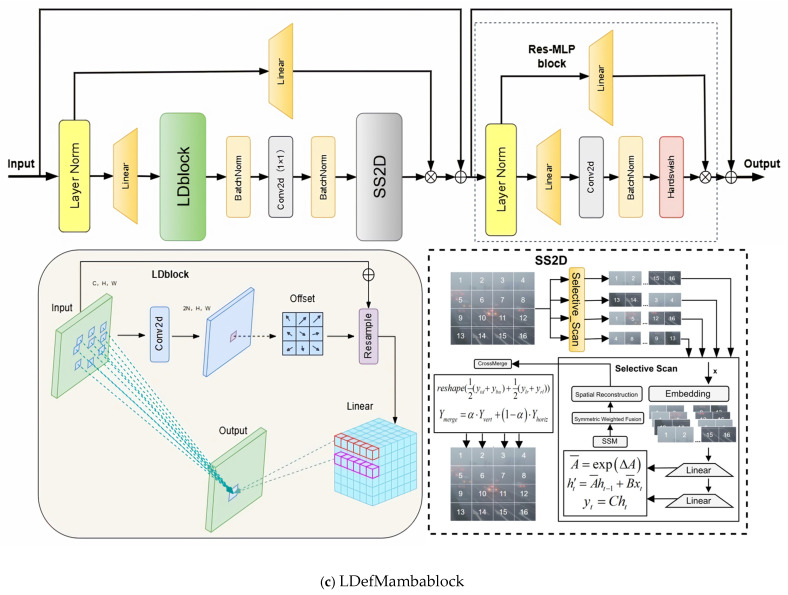
Overview of the method.

As shown in Figure 3a, our GMG-LDefmamba-YOLO framework as a whole follows the YOLO hierarchy and consists of three parts: backbone downsampling, neck feature fusion, and head detection. We replace the key small target extraction layer with two modules, GMGblock and LDefMambablock, and improve the neck feature pyramid structure to enhance the small target feature fusion ability. Figure 3b shows the GMGblock structure, which mainly improves the convolutional splicing method, extracts directional sensitivity features through two mirror-symmetrical four-way padded convolutional kernels, and combines the weighted fusion of Gaussian convolutional kernels with “heavy intermediate weights and decreasing surrounding weights” to strengthen the extraction ability of small target features. Figure 3c outlines the core structure of the LDefMamba block, the most critical components of which are the SS2D module, responsible for global feature extraction, and the LDblock module, responsible for local feature extraction at the downsampling layer. The module discards redundant parameters through the Mamba gating mechanism to reduce the computational cost.

In order not to destroy the basic feature extraction, we retain the original convolutional structure of the shallow network and maintain the Conv and C3k2 module design consistent with the foundation model at layers 0–2 to ensure that the preliminary information is not destroyed. In the middle layer feature extraction stage (p3/8 small target extraction layer), an improved LDefMambablock is introduced to pair the key small target features, and the feature splicing of the C3k2 improved feature pyramid is replaced by GMGblock at the key layer of the neck.

### 3.2. Gaussian Mask Gear Convolution GMGblock

In recent years, remote sensing small object detection methods based on convolutional neural networks (CNNs) have achieved excellent performance. However, these methods often employ standard convolution and ignore the spatial features of the pixel distribution of remote sensing small targets. In addition, small targets usually have a very low proportion of pixels and sparse feature information, resulting in weak feature expression ability and being extremely dependent on central features for positioning and detection. To solve this problem, we propose the GMGblock module, as shown in Figure 3b. The module combines multi-branch convolution to extract horizontal and vertical feature information from images through asymmetric filling and dynamic weighted fusion through Gaussian masks.

First, the input feature map is split into eight branches, and each four branches are used as a group to carry out two mirror-symmetrical four-way expansion convolutions, and the two sets of expansion convolutions are concatenated into a mirror-symmetric receptor field with the shape of a windmill through the splicing methods of “up, left, bottom, right” and “left, up, right, bottom”, respectively, and then the two groups of branches are concatenated to obtain a completely symmetrical gear-shaped receptive field with decreasing weights from the center to the periphery. Finally, the dynamically weighted multi-scale feature fusion is carried out in combination with the Gaussian mask mechanism to further strengthen the central feature. This method makes full use of the input feature map, strengthens the central feature extraction ability through its own multi-branch convolution and improved splicing method without introducing redundant information to increase the computational workload, and combines dynamic Gaussian weighting to match the central features of small targets to the greatest extent.

Specifically, the GMGblock module works as follows: first, the feature map $X^{\left(h_{1},w_{1},c_{1}\right)}$ with the number of input channels, c1 is divided into eight direction-sensitive branches, each with a thickness of c1/8, and then four of the branches are asymmetrically expanded (padding) in the direction of up, left, bottom, and right, and the other four branches are asymmetrically expanded in the direction of left, up, right, and bottom. This process is described as follows:

Vertical Upper Branch (Top):

Apply asymmetric padding $P^{\left(1,0,0,3\right)}$ to the input (left fill 1 pixel, right fill 0 pixels, top fill 0 pixels, bottom fill 3 pixels) to obtain the post-fill tensor $X_{P^{\left(1,0,0,3\right)}}^{\left(h_{1}+3,w_{1}+1,c_{1}\right)}$. Using 1 × 3 horizontal convolutional kernels $W_{1}^{\left(1,3,c^{\prime }\right)}$ for convolution, combined with batch normalization (BN) and SiLU activation, the following results are calculated:

Vertical Bottom Branch (Bottom):

Apply asymmetric padding $P^{\left(0,3,0,1\right)}$ to the input (left fill 0 pixels, right fill 3 pixels, top fill 0 pixels, bottom fill 1 pixel) to obtain the post-fill tensor $X_{P^{\left(0,3,0,1\right)}}^{\left(h_{1}+1,w_{1}+3,c_{1}\right)}$. Using 3 × 1 vertical convolutional kernels $W_{2}^{\left(3,1,c^{\prime }\right)}$ for convolution, combined with batch normalization (BN) and SiLU activation, the following results are calculated:

Horizontal Left Branch (Left):

Apply asymmetric padding $P^{\left(0,1,3,0\right)}$ to the input (left fill 0 pixels, right fill 1 pixel, top fill 3 pixels, bottom fill 0 pixels) to obtain the post-fill tensor $X_{P^{\left(0,1,3,1\right)}}^{\left(h_{1}+3,w_{1}+1,c_{1}\right)}$. Using 1 × 3 horizontal convolutional kernels $W_{3}^{\left(1,3,c^{\prime }\right)}$ for convolution, combined with batch normalization (BN) and SiLU activation, the following results are calculated:

Horizontal Right Branch (Right):

Apply asymmetric padding $P^{\left(3,0,1,0\right)}$ to the input (left fill 3 pixels, right fill 0 pixels, top fill 1 pixel, bottom fill 0 pixels) to obtain the post-fill tensor $X_{P^{\left(3,0,1,0\right)}}^{\left(h_{1}+1,w_{1}+3,c_{1}\right)}$. Using 3 × 1 vertical convolutional kernels $W_{4}^{\left(3,1,c^{\prime }\right)}$ for convolution, combined with batch normalization (BN) and SiLU activation, the following results are calculated:

(1) $$X_{1}^{\left(h^{\prime },w^{\prime },c^{\prime }\right)}=\mathrm{SiLU}\left(\mathrm{BN}\left(X_{P^{\left(1,0,0,3\right)}}^{\left(h_{1}+3,w_{1}+1,c_{1}\right)}⊗W_{1}^{\left(1,3,c^{\prime }\right)}\right)\right)$$

(2) $$X_{2}^{\left(h^{\prime },w^{\prime },c^{\prime }\right)}=\mathrm{SiLU}\left(\mathrm{BN}\left(X_{P^{\left(0,3,0,1\right)}}^{\left(h_{1}+1,w_{1}+3,c_{1}\right)}⊗W_{2}^{\left(3,1,c^{\prime }\right)}\right)\right)$$

(3) $$X_{3}^{\left(h^{\prime },w^{\prime },c^{\prime }\right)}=\mathrm{SiLU}\left(\mathrm{BN}\left(X_{P^{\left(0,1,3,0\right)}}^{\left(h_{1}+3,w_{1}+1,c_{1}\right)}⊗W_{3}^{\left(1,3,c^{\prime }\right)}\right)\right)$$

(4) $$X_{4}^{\left(h^{\prime },w^{\prime },c^{\prime }\right)}=\mathrm{SiLU}\left(\mathrm{BN}\left(X_{P^{\left(3,0,1,0\right)}}^{\left(h_{1}+1,w_{1}+3,c_{1}\right)}⊗W_{4}^{\left(3,1,c^{\prime }\right)}\right)\right)$$

where X∈R represent the feature map obtained by applying different convolutional kernels, the convolutional step s determines the size of the output feature map, satisfying, $h^{\prime }=\frac{h_{1}+p_{h}-k_{h}}{s}+1$ and $h^{\prime }=\frac{h_{1}+p_{h}-k_{h}}{s}+1$, $p_{h},p_{w}$ is the fill height, width; $k_{h},k_{w}$ is the height and width of the convolutional kernel, and the final output channel number meets $c^{\prime }=\frac{c_{1}}{8}$.

(5) $$X^{\prime }=\mathrm{concat}\left(x_{1},x_{2},x_{3},x_{4}\right)$$

By performing this, the 4 c/8 branches of each group are spliced to obtain the convolutional kernel of the two c/2 branches. Due to the above asymmetric expansion and dislocation splicing, each convolutional core has a mirror-symmetrical pinwheel-shaped model receptor field. Splice the two branches:

(6) $$X^{\prime \prime }=\mathrm{concat}\left(x^{\prime },x_{1}^{\prime }\right)$$

At this point, the number of channels is restored to $c^{\prime \prime }=c_{1}$, and the receptive field is perfectly symmetrical, showing a gear shape with decreasing weights from the center to the surrounding area. Then, we introduce the Gaussian mask enhancement mechanism to optimize the spliced feature map. Specifically, the Gaussian mask is generated by the preset Gaussian kernel function, and its kernel value distribution follows the Gaussian distribution law, with the center of the feature map as the origin, and shows exponential attenuation to the surrounding area, so as to strengthen the feature weight of the central region and suppress edge noise interference. The formula for generating Gaussian kernels is as follows:

(7) $$K(i,j)=\frac{1}{2\pi \sigma^{2}}exp(-\frac{{\left(i-c\right)}^{2}+{\left(j-c\right)}^{2}}{2\sigma^{2}})$$

where *c* is the core center coordinate, which σ is the standard deviation of the Gaussian function, which controls the weight decay rate. The Gaussian kernel acts on the splicing feature map $X^{\prime \prime }$ through the convolutional layer, the batch normalization (BN), and SiLU activation to output the enhanced Gaussian feature map.

We generate the kernel matrix by expanding Equation (7) into a K × K tensor. For example, when K = 3 and sigma = 1.0, the generated kernel matrix is:

(8) $$\left[\begin{array}{ccc} 0.0585 & 0.1247 & 0.0585\\ 0.1247 & 0.2653 & 0.1247\\ 0.0585 & 0.1247 & 0.0585 \end{array}\right]$$

This matrix is then repeated to match the input/output channel number of gaussian_conv (i.e., c2 channels), ensuring each feature channel receives consistent Gaussian weighting. In the forward method, we apply the pre-initialized Gaussian kernel to the concatenated 8-branch feature map—essentially performing element-wise multiplication between the kernel and local feature patches, followed by summation. This operation implements pixels with high kernel weights (near target centers) and weakens background pixels.

In order to balance the contribution of the original splicing features and the Gaussian-enhanced features, a dynamic fusion weight mechanism is designed: the fusion weights are calculated by the learnable parameters ∂ and the Softmax function, and the two types of features are weighted and summed:

(9) $$fused\_feat=\omega_{0}\cdot concat\_feat+\omega_{1}\cdot gaussian\_feat$$

where $\omega_{0}$ and $\omega_{1}$ are the normalized weights ($\omega_{0}+\omega_{1}=1$), the model can automatically adjust the values of the two according to the task requirements, and flexibly strengthen the response of key regions while retaining the diversity of multi-branch features.

Finally, the channel integration of the fusion features is carried out by concatenation, and the final feature map $c^{\prime \prime \prime }=c_{1}$ with the output dimension. This operation not only further compresses the feature dimension but also enhances cross-channel information interaction and ensures the strong responsiveness of the center small object feature and the integrity of the spatial context.

In summary, the GMGblock captures rich directional sensitivity features through multi-branch asymmetric convolution, combined with the dynamic enhancement mechanism of a Gaussian mask, to achieve accurate adaptation to the characteristics of “strong dependence on central features and sparse spatial distribution” of small remote sensing targets, which significantly improves the discrimination of small target features while suppressing background noise. At the same time, by replacing the basic convolution module in C3k2 = False, and C3k2 = True of the bottleneck, the improvement can be applied to the C3k2 structure of the neck, optimizing the feature extraction, multi-scale feature fusion, and feature splicing of the pyramid.

### 3.3. Linear Deformable Mamba Block LDefMambablock

In the field of visual remote sensing, different distances change the size and shape of the target. Complex backgrounds, such as buildings, clouds, or vegetation, further obscure the target. Mamba is capable of modeling long-range feature dependencies, which is essential for understanding the global context in remote sensing images. Existing SSM-based models often employ scanning strategies to ensure connectivity between different regions of the image. However, this approach significantly increases feature redundancy within SSM, resulting in high computational costs when processing high-resolution remote sensing images, making it a performance bottleneck. At the same time, in remote sensing detection scenarios, traditional convolution is difficult to adapt to target scale changes and complex background interference due to the limitations of fixed sampling shapes and cannot accurately focus on effective feature areas. The traditional deformable convolution is limited by the parameter growth mode of square complexity, and when the number of sampling points increases, the computational overhead increases significantly, making it difficult to balance performance and efficiency under the lightweight demand. Traditional feature fusion modules mostly use fixed weights or simple splicing strategies, which lack the ability to dynamically adapt features at different levels and scales, which can easily cause redundancy or loss of feature information.

In order to solve this problem, we propose an efficient linear deformable Mamba LDefMambablock module, as shown in Figure 3c, which integrates the SS2D spatial state dual model based on SSM to capture global dependencies and the improved linear deformable convolution to extract accurate local features of variable size and performs post-processing by gating MLP and complementing channel nonlinear interactions.

Specifically, the structure can be decoupled into three independent functional components: the linear deformable sampling LDblock, the spatial state dual model SS2D, and the residual gated Res-MLP block.

#### 3.3.1. LDblock

The working principle of the LDblock module is shown in Figure 3c, which is divided into three steps: initial sampling coordinate generation, dynamic offset adjustment and coordinate correction, and bilinear interpolation and feature aggregation.

The first is the initial sampling coordinate generation: for any number of parameters N, such as (3, 5, 7, etc.), the “regular grid + irregular expansion” strategy is used to generate the initial sampling coordinates $P_{n}$, and the basic integer division (base_int = round ($\sqrt{N}$)) to construct a regular grid framework, and then irregularly expand the remainder (mod_number = N % base_int) to ensure that the distribution of sampling points is stable and flexible. Mathematically, the initial coordinate generation follows the following process:

(10) $$\left\{\begin{array}{l} \mathrm{base}\_\mathrm{int}=round\left\lfloor \sqrt{N}\right\rfloor \\ row\_number=\left\lceil \frac{N}{base\_\mathrm{int}}\right\rceil \\ \mathrm{mod}\_number=N\%base\_\mathrm{int} \end{array}\right.$$

Here, $\left\lfloor \sqrt{N}\right\rfloor$ calculates the largest integer less than or equal to $\sqrt{N}$ to form the base of the regular grid; $\left\lceil \frac{N}{base\_\mathrm{int}}\right\rceil$ (ceiling operation) determines the number of rows needed to accommodate all sampling points, ensuring no points are omitted; N%base_int gives the remainder of N divided by base_int, which guides the irregular expansion of sampling points.

The regular grid part generates coordinates $\left(p\_n\_x,p\_n\_y\right)$ through torch.meshgrid, and the irregular expansion part supplements the sampling points corresponding to the remainder, and finally splices into a complete initial coordinate matrix $P_{n}\in R^{2N\times 1\times 1}$.

Then there is dynamic offset adjustment and coordinate correction: to adapt to the change in the shape of the target, the LDblock predicts the offset of the sampling point $\Delta P\in R^{B\times 2N\times H\times W}$ through an independent offset branch $\left(p\_conv\right)$ and fuses it with the initial coordinates to generate a dynamic sampling position: $P=P_{0}+P_{n}+\Delta P$ among them is the datum coordinate grid is $P_{0}$, which is determined by the input feature map size and step size. The offset learning adopts a gradient scaling strategy to avoid feature instability caused by excessive offset in the early stage of training. The corrected coordinate P should be limited to the range of the feature map by cropping to ensure the sampling effectiveness:

(11) $$P_{clip}=clamp\left(P,0,H-1\right)\times clamp\left(P,0,W-1\right)$$

Specifically, LDblock predicts the offset of the sampling point through an independent offset branch p_conv and fuses it with the initial coordinates to generate a dynamic sampling position. The dynamic sampling position ${\hat{P}}_{n}$ is calculated as follows:

(12) $$\left\{\begin{array}{l} {\hat{P}}_{n}=P_{n}+\alpha \cdot \Delta P\\ {\hat{P}}_{n}=clip\left({\hat{P}}_{n},0,H-1\right)⊗clip\left({\hat{P}}_{n},0,W-1\right) \end{array}\right.$$

Among them, the datum coordinate grid is $P_{0}$, which is determined by the input feature map size and step size. α=0.1 is a gradient scaling factor to avoid feature instability caused by excessive offset in the early stage of training, and $clip\left(\cdot \right)$ restricts coordinates to the valid range of the feature map (H and W are the height and width of the feature map, respectively) to ensure sampling effectiveness.

Finally, bilinear interpolation and feature aggregation: based on dynamic coordinates $P_{clip}$, bilinear interpolation is used to achieve feature resampling. For each sample point $\left(x,y\right)$, its feature value is represented by the weighting sum of the surrounding four pixels:

(13) $$x_{\mathrm{offset}}=\sum_{q\in \left(q_{u},q_{rb},q_{lb},q_{rt}\right)}x_{q}\cdot y_{q}$$

where *q* is the integer coordinate, $y_{q}$ is the interpolation weight, which is calculated by the distance between the sample point and the integer coordinate.

Subsequently, bilinear interpolation is performed to aggregate features. For the corrected sampling coordinate ${\hat{P}}_{n}=\left({\hat{x}}_{n},{\hat{y}}_{n}\right)$, the feature value $F\left({\hat{P}}_{n}\right)$ is obtained as follows:

(14) $$F\left({\hat{P}}_{n}\right)=\sum_{q\in \left\{q_{11},q_{12},q_{21},q_{22}\right\}}w_{q}\cdot F\left(q\right)$$

where *q* denotes the four integer coordinates surrounding ${\hat{P}}_{n}$, and the interpolation weight $w_{q}$ is calculated as follows:

(15) $$w_{q}=\left(1-\left|{\hat{x}}_{n}-x_{q}\right|\right)\times \left(1-\left|{\hat{y}}_{n}-y_{q}\right|\right)$$

$w_{q}$ ensures that pixels closer to ${\hat{P}}_{n}$ have a greater impact on the interpolated feature value, facilitating continuous feature sampling for small targets.

In summary, LDblock acts as the local feature extraction part of the LDefMambablock module through the co-design of linear parameter growth, arbitrary sampling shape, and dynamic offset adjustment.

#### 3.3.2. SS2D Module

The working principle of the SS2D module is shown in Figure 3c, which is divided into four steps: selective scanning, direction sequence alignment, symmetrical weighted fusion, and spatial reconstruction.

The first is selective scanning: the selective-scanning mechanism is the core component of the SS2D module to realize global dependency modeling, which converts 2D visual features into linear complexity sequence modeling problems through three-level processing of four-way symmetric scanning, state-space transformation, and feature aggregation. The core innovation of this mechanism is that the spatial structure of the image is transformed into a sequence of temporal relationships through directional decomposition, and the secondary complexity bottleneck of traditional self-attention is broken through with the linear temporal characteristics of the state space model (SSM), while retaining the spatial context association of visual features.

For the input feature map $x\in R^{B\times C\times H\times W}$, the SS2D mechanism first expands into sequence data in four symmetrical directions (up, down, up, right, right, and left) through cross-scan operations. Taking the horizontal direction as an example, the scanning process can be broken down as follows:

Top-Down: Expand line by line along the image row dimension from top to bottom, and generate a sequence $xs_{td}\in R^{B\times C\times \left(H\times W\right)}$; Bottom-Up: Reverse scan along the row dimension to generate a reverse sequence $xs_{bu}\in R^{B\times C\times \left(H\times W\right)}$; Left-Right: Expand the column from left to right along the column dimension to generate a sequence $xs_{lr}\in R^{B\times C\times \left(H\times W\right)}$; Right-Left: Reverse scan along the column dimension to generate a reverse sequence $xs_{rl}\in R^{B\times C\times \left(H\times W\right)}$.

The four-way sequence $xs=\left[xs_{td};xs_{bu};xs_{lr};xs_{rl}\right]\in R^{B\times 4\times C\times \left(H\times W\right)}$ is obtained by splicing, and the operation covers the entire area of the image through directional symmetry, providing a multi-perspective sequence representation for subsequent global dependency modeling. Mathematically, a scan in a single direction can be expressed as follows:

(16) $$xs_{dir}=Scan\left(x,dir\right)=\mathrm{flatten}\left(transpose\left(x,dir\right)\right)$$

Among them $dir\in \left[td,bu,lr,rl\right]$ is the flattening feature map flattening operation; transpose adjusts the dimension order according to the direction to realize row-by-row expansion.

For a single direction (taking top-down as an example), the scanning process can be mathematically expressed as follows:

(17) $$S_{dir}=\mathrm{flatten}\left(X\left(permute\left(0,2,1,3\right)\right)\right)$$

where $x\in R^{B\times C\times H\times W}$ is the input feature map (B is batch size, C is channel number, H is height, W is width), $permute\left(0,2,1,3\right)$ means we reorder the input feature map’s dimensions from $\left(B\times C\times H\times W\right)$ to $\left(B\times H\times C\times W\right)$, which places the height dimension (H, rows) before channels, so when flattened, features are traversed row-by-row from top to bottom, enabling the desired scanning direction. Through this method, we can reorder dimensions to enable row-wise top-down scanning, and $\mathrm{flatten}\left(\cdot \right)$ converts the 2D feature map into a 1D sequence $S_{dir}\in {\mathbb{R}}^{B\times C\times (H\times W)}$.

SS2D uses the zero-order hold ZOH method to discretize the continuous state space model and adapt it to sequence processing. The state transfer equation for a continuous system is as follows:

(18) $$h^{\prime }\left(t\right)=Ah\left(t\right)+Bx\left(t\right),y\left(t\right)=Ch\left(t\right)$$

where $A\in R^{N\times N}$ is the state transition matrix, $B\in R^{N\times 1}$ is the input weight matrix, and $B\in R^{N\times 1}$ is the output observation matrix.

The sequence in each direction is transformed by the state space model, and the state transition matrix and the input matrix are discretized by zero-order hold ZOH to satisfy the following:

(19) $$\bar{A}=\exp\left(\Delta A\right),\bar{B}={\left(\Delta A\right)}^{-1}\left(\exp\left(\Delta A\right)-I\right)\Delta B$$

$\bar{A}$ and $\bar{B}$ are the discretized state transition matrix and the input matrix, of which Δ is the timescale parameter and I is the unit matrix. This recursive process processes the four-way sequence channel by channel, generating a hidden state sequence containing global dependencies.

The second is direction alignment: the direction inverse transformation of the four-way output sequence $y_{td},y_{bu},y_{lr},y_{rl}\in R^{B\times C\times L}$ is performed to restore the spatial position correspondence.

Top-down direction: The scan order is row first (each row from row 1 to H row, each column from column 1 to column W). Bottom-Up direction: The scanning order is reverse (each row from row H to row 1, each column from column 1 to column W). Left-Right direction: The scanning order is column first (each column from column 1 to column H, each row from row W to row 1); Right-Left direction: The scanning order is reverse column order (each column from column H to column 1, each row from W row 1 to row); The mapping of sequence indexes t and spatial coordinates $\left(i,j\right)$ is satisfied in the order of up-down, down-up, left-right, and right-left:

(20) $$\begin{array}{l} t=\left(i-1\right)\cdot W+\left(j-1\right),i\in \left[1,H\right],i\in \left[1,W\right]\\ t=\left(H-i\right)\cdot W+\left(j-1\right),i\in \left[1,H\right],i\in \left[1,W\right]\\ t=\left(j-1\right)\cdot H+\left(i-1\right),i\in \left[1,H\right],i\in \left[1,W\right]\\ t=\left(W-j\right)\cdot H+\left(i-1\right),i\in \left[1,H\right],i\in \left[1,W\right] \end{array}$$

The third is symmetric weighted fusion: weighted fusion is used in both vertical and horizontal directions to eliminate directional ambiguity and enhance symmetrical regional response. The complementarity of the vertical direction (row scan) can suppress the row direction noise, and the complementarity of the horizontal direction (column scan) can suppress the column direction noise, and the vertical and horizontal fusion results are dynamically balanced by learnable parameters α. The fusion formula is as follows:

(21) $$\begin{array}{l} Y_{vert}=\frac{1}{2}\left(Y_{td}+Y_{bu}\right)\\ Y_{horiz}=\frac{1}{2}\left(Y_{lr}+Y_{rl}\right)\\ Y_{merge}=\alpha \cdot Y_{vert}+\left(1-\alpha \right)\cdot Y_{horiz} \end{array}$$

where $Y_{vert}$ is vertical weighted fusion, $Y_{horiz}$ is horizontal weighted fusion, and $\alpha \in \left[0,1\right]$ are learnable parameters, initialized to 0.5, optimized by backpropagation.

Finally, the fourth is spatial reconstruction: reshape the fused sequence into $R^{B\times C\times H\times W}$ dimensions to complete the spatial mapping of global features. Mathematically, the merge operation can be expressed as follows:

(22) $$y=CrossMerge(\{y_{dir}\})=reshape(\frac{1}{2}(y_{td}+y_{bu})+\frac{1}{2}(y_{lr}+y_{rl}))$$

The mathematical nature of this operation is linear compression of the spatial dimension, calculating the mean of the original feature map corresponding to each pixel in the target size (H′, W′) for each channel’s feature map:

(23) $$Y_{out}\left[b,c,i^{\prime },j^{\prime }\right]=\frac{1}{h\cdot w}\sum_{p=0}^{h-1}\sum_{q=0}^{w-1}Y_{merge}\left[b,c,\left\lfloor \frac{i^{\prime }\cdot H}{H^{\prime }}\right\rfloor +p,\left\lfloor \frac{j^{\prime }\cdot W}{W^{\prime }}+q\right\rfloor \right]$$

where *b* is the batch size, c is the number of channels, $i^{\prime }$ and $j^{\prime }$ are the spatial coordinates on the output feature map, H and W are the spatial size of the sequence after fusion, and $H^{\prime }$ and $W^{\prime }$ are the target size of the output feature map after spatial reconstruction.

The process suppresses noise through directional symmetry while retaining global dependencies, so that the output feature map y not only contains local feature information, but also captures long-distance spatial correlations.

#### 3.3.3. Res-MLP Block

The working principle of the residual gated MLP module is shown in Figure 3c. Firstly, the linear transformation of the channel dimension is performed on the input feature map, and it is projected into a temporary high-dimensional feature space. Suppose the input feature map is $X\in R^{B\times C\times H\times W}$ (where *B* is the batch size, *C* is the number of channels, and *H* and *W* are the height and width, respectively), and the dimensional upscaling of the channel dimension is carried out through a fully connected layer to obtain the feature map $Y_{1}\in R^{B\times C_{mid}\times H\times W}$, where in $C_{mid}=C\times r$ (*r* is the expansion ratio, e.g., *r* = 2). This operation aims to provide more feature combination possibilities for subsequent nonlinear transformations and explore complex dependencies between channels.

Next, $Y_{1}$ is performed for nonlinear transformation on the Hardswish activation function, which is expressed as follows:

(24) Hardswish(x)=x⋅max(0,min(1,(x+3)/6))

This activation function, Hardswish, has significant advantages in remote sensing small target detection: for scenes where the proportion of small target pixels is extremely low and the eigenvalue is generally small, the linear segmentation feature of Hardswish in the interval $x\in \left[-3,3\right]$ can effectively amplify the weak feature signal (such as small target edges and local textures) and avoid feature loss caused by the saturation of the activation function.

After that, a fully connected layer is used to project the feature map from the high-dimensional intermediate space back to the original number of channel dimensions, obtaining $Y_{3}\in R^{B\times C_{mid}\times H\times W}$. At this time, the original feature map $Y_{3}$ are added element-by-element with X to form a residual connection. The purpose of this step is to alleviate the problem of gradient disappearance, so that the model can better transmit information in the deep structure, and retain the key features of the original input, which is the final output feature map $Y_{out}=X+Y_{3}$.

The gating mechanism plays an important role in the entire process. In the improved residual gating MLP module, a gating unit is introduced before the residual is attached. It divided $Y_{3}$ into two parts through a linear transformation, one part generates a gating signal $G\in {\mathbb{R}}^{B\times C\times H\times W}$ through an activation function (the element value in *G* is between 0 and 1, which is used to control the degree of passage of information), and the other part multiplies *G* element by element, and then adds it to *X*. In this way, the model can adaptively determine how much information from the original input X is retained and how much new information is introduced from the MLP-processed features $Y_{3}$ through gating signal control, further improving the model’s ability to select and fuse different features, and enhancing its ability to express complex data patterns, especially in tasks such as remote sensing and small object detection that require accurate capture of subtle features and are sensitive to background noise interference. It can better focus on the target features, suppress useless information in the background, and achieve the effect of lightweight.

## 4. Experiments

This section describes extensive experiments to evaluate the effectiveness and performance of this model in remote sensing target detection. First, the datasets used in the experiment are briefly introduced. Next, explain the experiment setup and evaluate the metrics. Finally, the results of ablation studies and comparative experiments are provided, followed by an analysis of the observed phenomena and trends.

### 4.1. Dataset

The object detection dataset DOTA-v1.0 [40] is a large-scale object detection dataset for optical remote sensing imagery, which includes 2806 images with a total of 188,282 object instances annotated with orientation bounding boxes. Sizes range from 800 × 800 to 4000 × 4000. These objects are divided into 15 categories: airplanes, baseball fields, bridges, track and field fields, small vehicles, large vehicles, ships, tennis courts, basketball courts, storage tanks, soccer fields, ring roads, ports, swimming pools, and helicopters. Image sizes in DOTA datasets range from 800 × 800 to 4000 × 4000 pixels. Each image in the dataset contains an average of 3 objects.

The Vehicle Detection in Aerial Imagery (VEDAI) dataset [41] is a comprehensive dataset specifically tailored for aerial imagery vehicle detection. A total of 1553 images were included. The images contain seven different categories and a total of 3575 object instances. These objects are divided into nine categories: cars, trucks, boats, tractors, campers, pickups, airplanes, others, and vans. The dataset offers images in 512 × 512 and 1024 × 1024 resolution formats. The dimensions of objects are primarily concentrated in the range of 12 × 12–24 × 24 pixels, and each image resolution includes data for both visible and infrared image registration, catering to the diverse needs of image analysis. Each image in the dataset contains an average of 3 objects.

USOD is a dataset based on UNICORN 2008 [42] for detailed manual annotation of small and medium-sized vehicles. The training set has 3000 images in sizes of 416 × 416 and 640 × 640. Each image contains an average of 14.5 objects, with the dimensions of the objects mainly concentrated in the range of 8 × 8–16 × 16 pixels, and each image in the dataset contains an average of 14.5 objects.

The details of DOTA-v1.0, VEDAI, and USOD are shown in Table 1.

The datasets used in this study are publicly available from their official repositories:

DOTA-v1.0 at https://captain-whu.github.io/DOTA/dataset.html (accessed on 2 July 2025) under the CC BY-NC-SA 4.0 License.

VEDAI can be accessed at https://downloads.greyc.fr/vedai/ (accessed on 2 July 2025) under a custom non-commercial license for academic research.

USOD can be accessed at https://github.com/AFRL-RY/data-unicorn-2008 (accessed on 2 July 2025) under the MIT License.

### 4.2. Implementation Details and Evaluation Metrics

In our experiments, we evaluate the directional object detection model using three datasets: DOTA-v1.0, VEDAI, and USOD. To ensure fair comparisons, we adhere to the dataset processing methods used in other mainstream studies [43] while strictly avoiding train/val overlap to eliminate data leakage risks. For DOTA-v1.0, we first followed its official split [40] to retain the original training set (1411 images) and validation set (458 images) without any cross-subset mixing. We then performed multi-scale cropping in a subset-exclusive manner: we scaled the original images of the training set at three ratios (0.5, 1.0, 1.5) and cropped each scaled image into 1024 × 1024 patches, and independently applied the same scaling and cropping rules to the validation set images, with no data interaction between the two subsets. After cropping, we added a spatial overlap check: we calculated the intersection-over-union (IoU) of spatial coordinates (relative to the original image) between each training patch and validation patch, discarding and re-cropping patches with IoU > 0.1 to avoid meaningful feature overlap. We also tracked the source ID of each original image for all cropped patches, confirming no validation-set parent IDs appeared in the training patch pool and vice versa. Through these measures, we finally obtained 15,749 training patches and 5297 validation patches, ensuring the integrity of small target features (target pixel size mainly concentrated in 20 × 20–300 × 300 pixels) while maintaining strict dataset separation.

For the VEDAI dataset, all category annotations in the dataset are retained to maintain the original data distribution characteristics, in which the target pixel size is mainly concentrated in the range of 12 × 12–24 × 24 pixels, and the small target presents a natural distribution state in the complex background. A total of 1224 training sets and 309 verification sets were obtained.

For the USOD dataset, the training set and validation set images are sorted into two sizes: 416 × 416 and 640 × 640, and the original distribution features of small targets (8 × 8–16 × 16 pixels) in the dataset are maintained. A total of 2100 training sets and 900 verification sets were obtained.

The experiment was conducted on Windows 11. The deep learning environment is equipped with CUDA 12.6 and the Pytorch 2.6.0 framework, trained on NVIDIA RTX4060 GPUs. The YOLOv11 algorithm scales based on network width and depth, including multiple variants: YOLOv11n, YOLOv11s, YOLOv11m, YOLOv11 l, and YOLOv11x. As a starting point, YOLOv11n is selected as a baseline, followed by network improvements.

To compare with SOTA results and ensure the comparability and validity of experimental results, we unified the training epoch count for both main experiments and ablation studies to 300 epochs. To enrich the input image, the initial learning rate is initialized to 0.01 using Mosaic enhancement technology, and the SGD optimizer is selected to optimize the total loss function. The training batch size is set to eight. The momentum parameter used is 0.937, and box_loss (bounding box loss), cls_loss (classification loss), and dfl_loss (Distribution FocalLoss) were used as loss functions of YOLOv11. To mitigate overfitting, an early stop method was implemented during training. For evaluation, our experiments utilized standard deep learning metrics such as precision (*P*), recall (*R*), average accuracy (*AP*), per-image predicted speed, and the memory of the model.

The formula for calculating accuracy is as follows:

(25) $$\begin{array}{l} P\;=\frac{N_{TP}}{N_{TP}+N_{FP}} \end{array}$$

The formula for calculating the recall rate is as follows:

(26) $$R\;=\frac{N_{TP}}{N_{TP}+N_{FN}}$$

The formula for calculating average accuracy is as follows:

(27) $$AP=\int_{0}^{1}P\left(R\right)dR$$

where *P*(*R*) is the precision–recall curve formula.

Our experimental evaluation metrics include average accuracy (*mAP*), model parameter count (parameters), and model computational complexity (FLOPs).

*mAP* is a comprehensive metric obtained by the average *AP* values. It uses an integral method to calculate the area under the precision–recall curve for all categories. Therefore, *mAP* can be calculated as follows:

(28) $$mAP=\frac{AP}{N}$$

where *N* is the number of categories.

The parameters and FLOPs are calculated by providing the same batch of images to the model. A parameter refers to the total number of parameters in a model, while FLOP measures computational complexity, representing the number of floating-point operations required to process a given input.

### 4.3. Ablation Study

Our proposed GMG-LDefmamba-YOLO consists of two key components: the GMGblock and the LDefMambablock. To verify the effectiveness of these components and ensure the rigor and generalizability of experimental results, we conducted ablation experiments with YOLOv11 [24] as the baseline model and simultaneously selected the above three representative remote sensing datasets for testing. All ablation experiments followed a unified protocol (300 training epochs, SGD optimizer with momentum 0.937, batch size 8).

#### 4.3.1. Analysis of the GMGblock on Branch Design and Gaussian Mask

**Figure 4:**
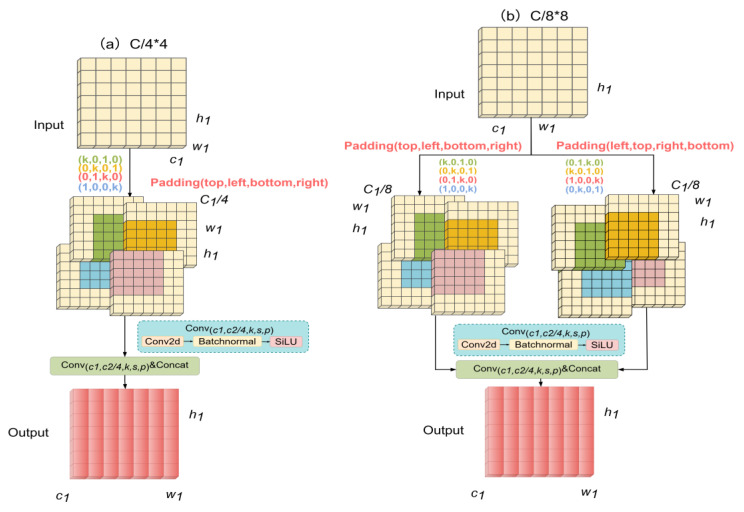

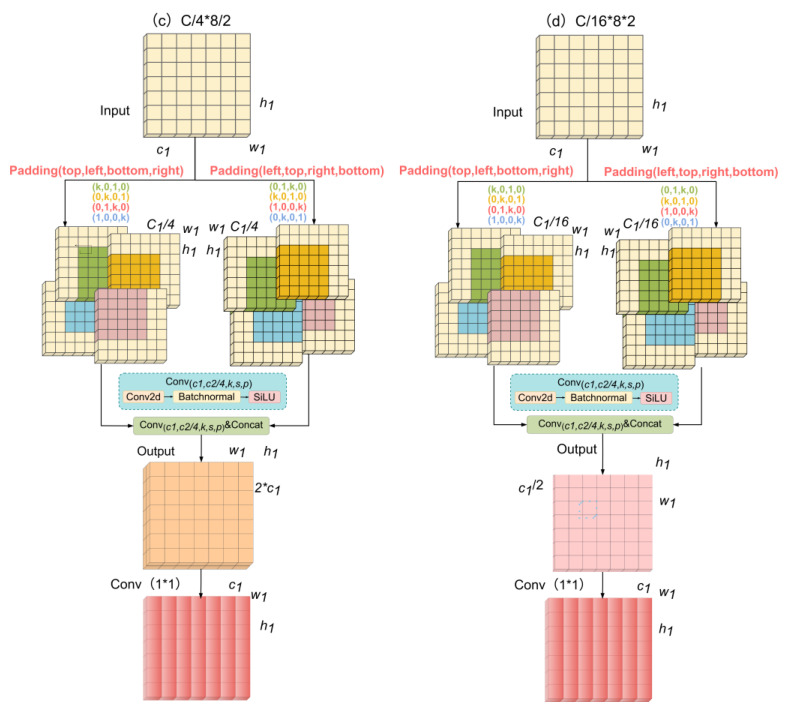
Schematic diagram of different branches of the GMGblock module.

As shown in Table 2, we studied the kernel design of the branch splicing strategy in GMGblock. C/4*4 is a four-branched asymmetric structural receptor field (half of the gear). C/4*8/2 is the number of channels per branch, C/4, and the eight-branch splicing is 2C, and then compressed into 1C by 1 × 1 convolution. C/16*8*2 is the number of channels per branch C/16, and the eight-branch splicing is C/2, which is restored to 1C through 1 × 1 convolution. C/8*8 is the eight-branch symmetric convolutional splicing mentioned in the text as the gear-shaped receptive field. As shown in Figure 4, the design diagram of different branches of the GMGblock module is shown, where ‘a’ corresponds to C/4*4, ‘b’ corresponds to C/8*8, ‘c’ corresponds to C/4*8/2, and ‘d’ corresponds to C/16*8*2.

The results show that eight-branch convolution enhances symmetry and achieves better accuracy than four-branch convolution. Among them, the C/8 × 8 branching strategy attains the best performance: on USOD, it reaches 90.35% mAP50 with 68 FLOPs and 16.88 M parameters; on DOTA-v1.0, it obtains 70.91% mAP50 with 79 FLOPs and 18.59 M parameters; and on VEDAI, it achieves 77.94% mAP50 with 77 FLOPs and 17.94 M parameters. The C/16 × 8 × 2 branching strategy has the lowest computational cost (e.g., 76 FLOPs on USOD) but poor accuracy (89.77% mAP50 on USOD). The C/4 × 8/2 branching strategy neither performs well in speed nor accuracy (e.g., 80 FLOPs and 89.98% mAP50 on USOD), which is speculated to be due to the increased computational load from more channels and the loss of some transmission characteristics during 1 × 1 convolutional compression. These findings suggest that the C/8 × 8 branching strategy achieves the best trade-off between speed and precision, maximizing mAP50 while keeping FLOPs at a reasonable level. It effectively splices the symmetrical gear-shaped receptive field while avoiding excessive computational overhead, making it an optimal choice for balancing small target detection accuracy and inference efficiency across multiple datasets. At the same time, we note that the USOD dataset FLOPs are significantly smaller than VEDAI’s because the USOD dataset image size is smaller (training set image size 416 × 416 and 640 × 640), there are fewer target species (single-class detection only), and the target size is smaller (mainly concentrated in 8 × 8–16 × 16 pixels), while the VEDAI dataset image has a resolution of 512 × 512 and 1024 × 1024, and the target size is relatively larger, and the model needs to detect three types of targets at the same time, and the characteristics of these datasets themselves make the computational amount significantly different.

In addition, it is also crucial to explore the effects of isolating the Gaussian mask alone. As shown in Table 3, We constructed two variants of the GMGblock for comparison on datasets: GMGblock w/o Gaussian mask: This variant retains the C/8*8 branch design (verified as optimal in previous experiments) but removes the Gaussian mask operation. Feature maps are processed only by the gear-shaped convolutional branches without spatial attention weighting from the Gaussian mask.

Experiments show that equipping with a Gaussian kernel improves model accuracy while causing only a minimal speed overhead. It is speculated that this is because the Gaussian kernel is statically defined and directly weighted. This kind of ‘weight overlay’ processing usually only brings linear-scale complexity and does not cause a speed burden. This proves the adaptability of the Gaussian mask block to this improvement.

#### 4.3.2. Analysis of the GMGblock on Kernel Sizes

GMGblock uses a horizontal convolutional kernel with a kernel size of 1 × k and a vertical convolutional kernel with a kernel size of k × 1 in an eight-branch expanded convolution, which directly affects the range of the receptive field and the ability to capture small target features. In order to verify the influence of kernel size on remote sensing small target detection, we took these datasets as the test object, fixed the branching strategy as the optimal C/8 × 8, only adjusted the convolutional kernel size (k = 3, 5, 7, 11), and compared the model parameters (Paras), computational power (FLOPs), and detection accuracy (mAP50) under different configurations, and the results are shown in Table 4.

The results show that the convolutional kernel size of 3 (1 × 3 and 3 × 1) has the best detection performance for remote sensing small targets in GMGblock. On USOD, it achieves 90.35% mAP50 with 68 GFLOPs and 16.88M parameters; on DOTA-v1.0, 70.91% mAP50 with 79 GFLOPs and 18.59 M parameters; and on VEDAI, 77.94% mAP50 with 77 GFLOPs and 17.94 M parameters. Its receptive field (about 40–60 pixels) matches the size of remote sensing small targets. Although increasing the kernel size can cover a wider range, it introduces redundant background information, increases the computational burden, and reduces the discriminability of small target features. When the convolutional kernel size is 5, redundant background is introduced, and mAP50 drops to 89.45% (USOD), 69.89% (DOTA-v1.0), and 76.85% (VEDAI). When the kernel size is 7, the receptive field is too large, leading to feature dilution, with mAP50 at 89.98% (USOD), 70.32% (DOTA-v1.0), and 77.00% (VEDAI). When the kernel size is 11, the coverage is excessively wide, causing spatial sparsification, and mAP50 is only 87.62% (USOD), 69.40% (DOTA-v1.0), and 76.13% (VEDAI), while computational volume surges (e.g., 83 GFLOPs and 26.44 M parameters on USOD). This verifies the rationality of GMGblock’s adaptation to the characteristics of small remote sensing targets and conforms to the design logic of “heavy central weight”.

#### 4.3.3. Analysis of the LDefMambablock on Necessity of Each Component

**Figure 5:**
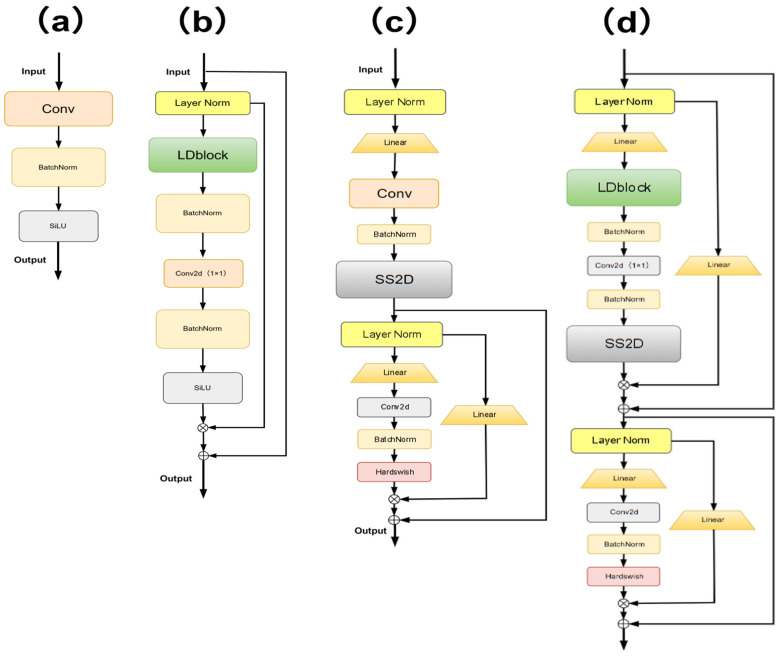
Schematic diagram of the integration of different components. Among them, (**a**) is the base convolution, (**b**) integrates LDblock on the basis of (**a**), (**c**) integrates SS2D and MLP on the basis of (**a**), and (**d**) is our LDefMambablock structure in this paper.

As mentioned earlier, LDefMambablock can be decoupled into three independent functional components: the linear deformable sampling LDblock, the spatial state dual model SS2D, and the residual gated MLP module. Among them, MLP has been shown to be used in conjunction with SS2D structures as a conditioning of the Mamba gating mechanism. In this section, we independently examine each module in LDefMambablock, and before proving the necessity of individual components, we downsample using conventional convolution to assess the impact of component performance on accuracy. We use the USOD dataset as the test object. As shown in Table 5, we have replaced the relevant modules on the original YOLOv11 with LDefMambablock. a, b, c, and d are different component forms of LDefMambablock, and we only study the construction of different LDefMambablocks to verify the effectiveness of each component. The “√” proof module in the table integrates the components, and the “×” represents that the components are replaced by standard convolutions. The schematic diagram of the different structures of the alternative is shown in Figure 5.

From the results, when LDefMambablock does not integrate any components (LDblock, SS2D, and MLP are all replaced by standard convolution, corresponding to structure “a”), the model achieves 88.87% mAP50 with a speed of 3.5 ms and memory usage of 1257 MB. When only LDblock is integrated (structure “b”), mAP50 increases to 89.59% (speed: 3.6 ms, memory: 1289 MB), indicating the effectiveness of linear deformable sampling for local feature capture. When SS2D and MLP are integrated (structure “c”), mAP50 is 89.83% (speed: 3.9 ms, memory: 1365 MB), demonstrating the effectiveness of global dependency modeling combined with the gating regulation mechanism for background feature discrimination. When the three components are integrated (structure “d”), mAP50 reaches the highest at 90.35% (speed: 4.2 ms, memory: 1418 MB), which is significantly higher than that of other combinations. This verifies the necessity of the synergy of LDblock’s local features, SS2D’s global dependency, and MLP’s gating regulation, and effectively improves the remote sensing small target detection performance through complementary mechanisms, while the increase in speed and memory is within a reasonable range for the performance gain.

### 4.4. Comparisons with Previous Methods

In order to further test the effectiveness of GMG-LDefmamba-YOLO, it was compared with the original YOLOv11 algorithm and other mainstream detection methods, including DSSD [44], RefineDet [45], YOLOv3 [46], YOLOv5 [47], YOLOv8 [48], YOLOv11 [24], YOLOv12 [49], YOLOv13 [50], Gold-YOLO [51], BGF-YOLO [52], FFCA-YOLO [53], DINO [54], DNTR [14], RFLA [55], Mamba-YOLO [21], FBRT-YOLO [56], etc. Both DSSD and RefineDet are classic and efficient object detectors. RefineDet stands out as a hybrid structure that combines the advantages of both primary and two-level object detection methods. Meanwhile, DSSD is an enhanced version of SSDs specifically designed to improve the detection accuracy of small objects. YOLOv3, YOLOv5, YOLOv8, YOLOv11, YOLOv12, and YOLOv13 are the cornerstone algorithms in the YOLO family, while Gold-YOLO, BGF-YOLO, and FFCA-YOLO represent advancements built on the YOLO framework. Gold-YOLO and BGF-YOLO enhance the network’s multi-scale feature fusion capabilities, while FFCA-YOLO improves the detection of small objects. DINO and DNTR are based on the transformer architecture, which has been improved for remote sensing object detection. RFLA is a label assignment method tailored for the same purpose. The comparison algorithm used in our study, RFLA, is built on top of Cascade R-CNN. DNTR, RFLA, TPH-YOLOv5, and FFCA-YOLO are all recently published remote sensing object detection algorithms. In addition, Mamba-YOLO and FBRT-YOLO are the latest remote sensing object detection YOLO algorithms released in 2025, of which Mamba-YOLO is the first algorithm to combine the Mamba algorithm with YOLO and achieve SOTA effect, and FBRT-YOLO is a recent further exploration and improvement for remote sensing small target detection.

**Figure 6:**
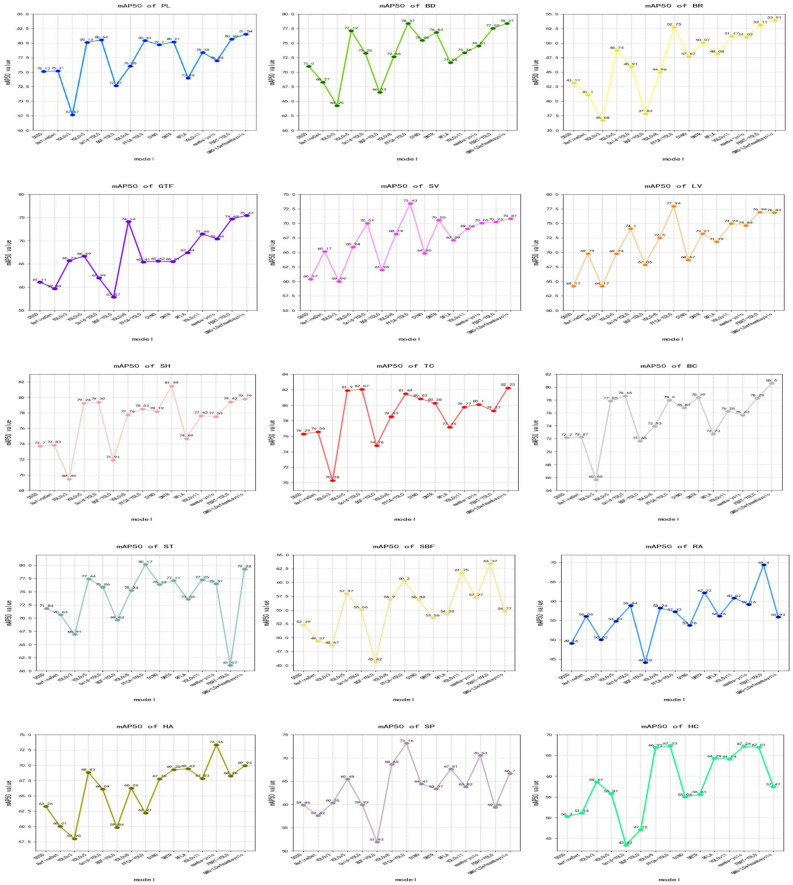
Average detection accuracy of the DOTA-V1.0 dataset. Each point represents the accuracy of the comparison models in a given category, where the horizontal axis represents the different models and the vertical axis represents the mAP50 value for each category.

**Figure 7:**
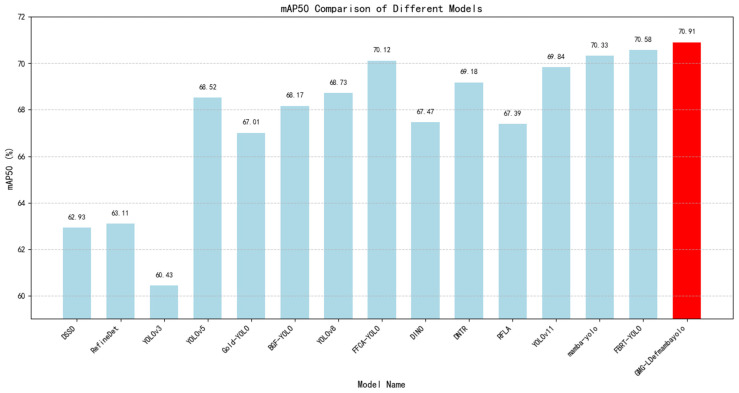
GMG-LDefmamba-YOLO is represented by red bars and achieves the highest detection accuracy of 70.91% on this dataset. Average detection accuracy across all categories in the DOTA-V1.0 dataset.

**Figure 8:**
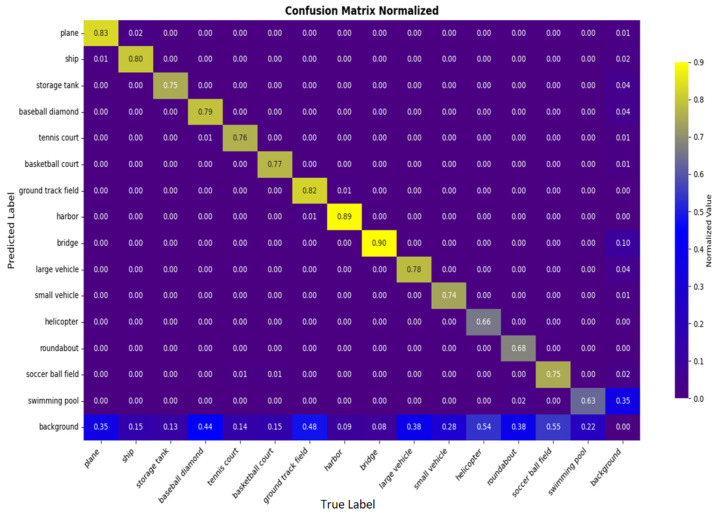
Confusion matrix for the DOTA-V1.0 dataset.

DOTA-v1.0 experimental results: We compare GMG-LDefmamba-YOLO with the above mainstream detection methods on the DOTA-v1.0 dataset, and the reported DOTA results are obtained by submitting the prediction results to the official evaluation server. As shown in Table 6, our GMG-LDefmamba-YOLO achieves state-of-the-art performance—its mAP50 reaches 70.91%, which is 0.58 percentage points higher than Mamba-YOLO, 0.33 percentage points higher than FBRT-YOLO, 0.48 percentage points higher than YOLOv12, and 0.36 percentage points higher than YOLOv13; it also achieves an excellent mAP50:95 of 50.39%, outperforming Mamba-YOLO by 0.63 percentage points, FBRT-YOLO by 0.46 percentage points, YOLOv12 by 2.61 percentage points, and YOLOv13 by 0.76 percentage points. This proves the effectiveness and efficiency of the proposed GMG-LDefmamba-YOLO model, which shows high recognition ability and stable localization performance for complex objects in remote sensing images. Figure 6 and Figure 7 show a line chart of detection accuracy by category and a bar chart of overall detection accuracy, respectively. Figure 8 shows the confusion matrix generated by the model on the DOTA-v1.0 test set, where the rows represent the true category and the columns represent the predicted class. The diagonal elements from the top left to the bottom right indicate the accuracy of the model in correctly classifying each category.

**Figure 9:**
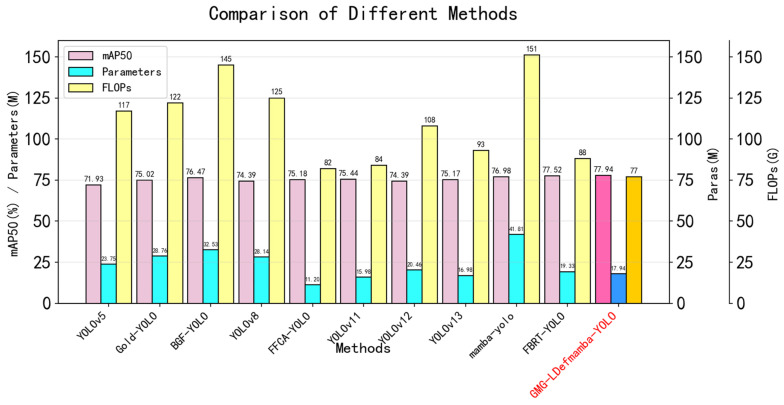
Bar charts comparing mAP50, parameters, and FLOPs metrics on the VEDAI dataset. The results of our method are prominently colored.

Experimental results of VEDAI: The reduction in target pixels in VEDAI is extremely significant, and the types of targets are diverse, which poses a challenge to the robustness and generalization of model performance. We evaluated the performance of GMG-LDefmamba-YOLO on the VEDAI dataset based on nine state-of-the-art methods. As shown in Table 7, both the mAP50 and FLOPs of GMG-LDefmamba-YOLO were optimal, ranking third in terms of parameter amount, second only to the YOLOv11 baseline model. This shows that the limited gating mechanism combined with MAMBA affects the computational amount of the YOLO hierarchy but greatly improves the detection performance and detection speed. Figure 9 shows a bar chart comparing mAP50, parameters, and FLOPs metrics on the VEDAI dataset.

Experimental results of USOD: The instances in the USOD dataset are of a single type, and the target size is very small; the average number of targets per image is large, which tests the model’s small target detection ability, dense target discrimination ability, and detection efficiency. Table 8 compares the 9 state-of-the-art methods for precision, recall, mAP50, speed, and memory on the USOD dataset. GMG-LDefmamba-YOLO outperformed previous methods, confirming the excellent small object detection capability of our improved model.

Additionally, breaking down detection accuracy by object scale is critical for validating the effectiveness of our method—especially for remote sensing small target detection, where performance differences across scales directly reflect the model’s ability to address the core challenge of “small target feature sparsity”. To solidify the claims, we conducted scale-specific accuracy analysis as shown in Table 9. The minimum bounding box (MBB) area of the target is defined as the smallest axis-aligned rectangle that can completely enclose the target area in the remote sensing image. Aligned with remote sensing target detection standards (following the DOTA dataset’s scale division and industry common practices), we divided object scales into four categories based on the minimum bounding box (MBB) area of targets in the test datasets (DOTA-v1.0, VEDAI, USOD): small targets (MBB area < 32 × 32 pixels, e.g., small vehicles, streetlights in DOTA-v1.0; 8 × 8–16 × 16 pixel targets in USOD), medium targets (32 × 32 ≤ MBB area < 96 × 96 pixels, e.g., ships, airplanes in DOTA-v1.0; 20 × 20–24 × 24 pixel targets in VEDAI), large targets (96 × 96 ≤ MBB area < 256 × 256 pixels, e.g., buildings, bridges in DOTA-v1.0), and extra-large targets (MBB area ≥ 256 × 256 pixels, e.g., large-scale roads, industrial facilities in DOTA-v1.0). The results demonstrate that our method effectively improves detection accuracy for objects of different scales, proving that this improvement, achieved by combining ‘global and local’ features, enhances object detection capabilities in the field of remote sensing.

Overall, the experimental results show that the proposed GMG-LDefmamba-YOLO method not only achieves excellent performance on the DOTA-V1.0 dataset but also shows excellent detection performance on the VEDAI and USOD datasets, especially in small target-dense scenarios. Our GMG-LDefmamba-YOLO can significantly improve the detection accuracy of remote sensing targets, mainly due to the following reasons: (1) The proposed GMGblock module constructs a gear-like receptive field with “heavy central weight and decreasing peripheral weight” through mirror-symmetrical four-way padding convolution and Gaussian mask mechanism, which accurately adapts to the characteristics of small targets “dependent on central feature and sparse spatial distribution”, and enhances the discriminatory feature of small targets. (2) The designed LDefMambablock module integrates LDblock and SS2D components: LDblock realizes flexible local feature capture through linear deformable sampling and adapts to target scale changes; SS2D effectively balances detection accuracy and computational efficiency through four-way symmetric scanning and state-space transformation while maintaining linear computational complexity while modeling global dependence. (3) The improved neck feature pyramid structure replaces the traditional convolutional module with GMGblock, which strengthens the multi-scale feature fusion capability and especially improves the transfer and aggregation effect of small target features in deep networks.

**Figure 10:**
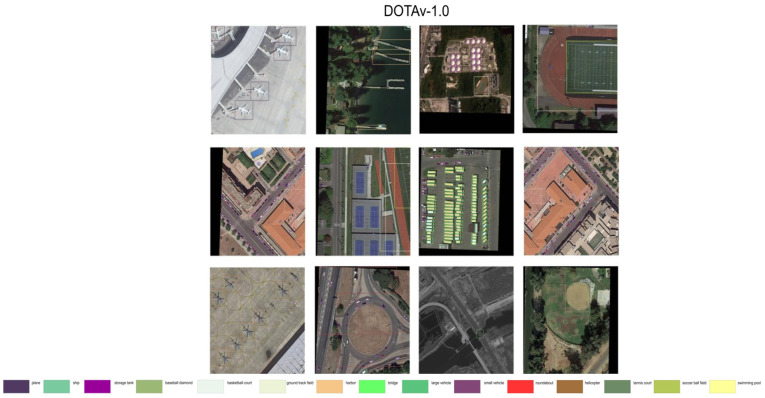
Partial visualization of the results of the method on the DOTAv-1.0 dataset.

**Figure 11:**
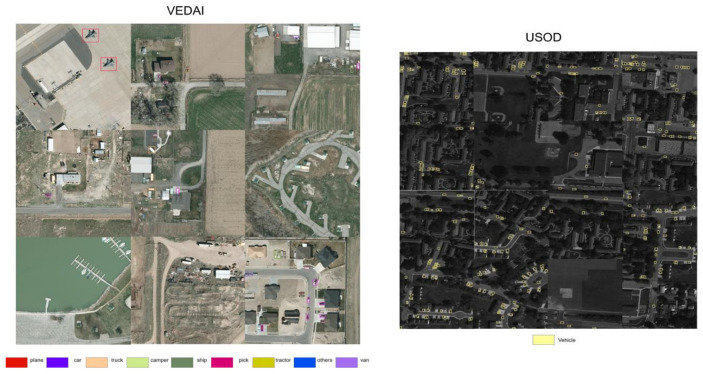
Partial visualization of the results of the method on the VEDAI and USOD datasets.

The results of the DOTAv-1.0. dataset, VEDAI dataset, and USOD dataset detection visualization are shown in Figure 10 and Figure 11.

**Figure 12:**
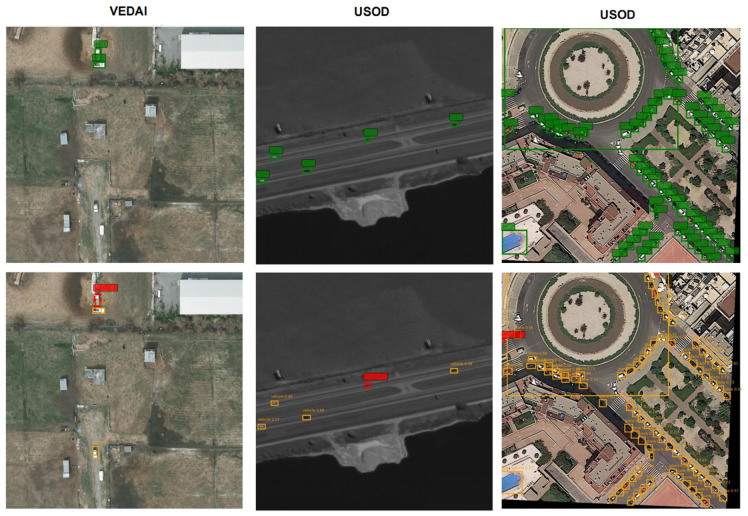
False positives and missed detections of DOTAv-1.0, VEDAI, and USOD. The green border indicates the correct labeling scheme, the yellow border indicates the targets correctly recognized by the model, and the red border indicates false positives and missed detections.

To comprehensively understand the limitations of our proposed model and address potential robustness concerns, we conducted a detailed failure analysis. We specifically focused on identifying worst-case scenarios, including high-confidence false positives and challenging missed detections (Figure 12).

As the results show, our model faces challenges in specific scenarios. For example, white vehicles in VEDAI scenes and black vehicles in USOD scenes are prone to missed detections—this is directly tied to their high similarity to surrounding backgrounds: white vehicles blend with white road shoulders (low color contrast), while black vehicles match the dark texture of farmland (consistent grayscale distribution). Additionally, false positives occur when the model misidentifies background textures (e.g., grid-like farmland ridges in USOD, light-colored road markings in VEDAI) that share structural patterns with target features (e.g., small vehicle outlines). These situations suggest that while our model performs well overall, further refinement could involve enhancing the feature discrimination ability, perhaps by incorporating more context-aware mechanisms to better distinguish targets from similar background elements, so as to boost its performance in such edge cases.

## 5. Conclusions

In this paper, an improved model based on the YOLOv11 framework, GMG-LDefmamba-YOLO, is proposed to solve the core challenges of remote sensing image target recognition, such as dense distribution, feature sparseness, drastic scale changes, and complex background interference, including two core modules, GMGblock and LDefMambablock. The traditional YOLO framework is refined from the perspectives of convolutional splicing, deformable convolutional sampling, selective scanning mechanism, global modeling of spatial state dual modeling, and residual gating lightweighting.

GMGblock forms a gear-like symmetric receptive field through mirror-symmetrical eight-way padding volume branching and combines Gaussian mask dynamic modulation feature weights to enhance the extraction of small target center features and suppress complex background interference. LDefMambablock integrates linear deformable sampling (LDblock), spatial state dual model (SS2D), and residual gating MLP components, which combine the advantages of flexible local feature capture and global dependence efficient modeling, dynamically adapt to target scale changes and spatial distribution, and reduce the amount of computational parameters to achieve lightweight performance. Experiments on DOTA-v1.0, VEDAI, and USOD datasets have shown that the mAP50 of GMG-LDefmamba-YOLO reached 70.91%, 77.94%, and 90.28%, respectively, compared with baseline YOLOv11 and mainstream studies. The methods (e.g., Mamba-YOLO, FBRT-YOLO, etc.) are improved while maintaining the lightweight characteristics of 17.94 M parameters and 77 FLOPs, which is lower than the computational cost of some comparison models. Comparative experiments show that the model achieves a better balance between detection accuracy and parameter efficiency. The visualization results further confirm that GMG-LDefmamba-YOLO significantly improves the detection confidence in dense small targets, complex backgrounds, and multi-scale scenarios, while reducing computational complexity and computing power requirements, providing efficient and reliable technical support for image analysis in remote sensing monitoring, UAV inspection, and other fields.

In this study, although the GMG-LDefmamba-YOLO network shows superior detection performance, it has limited improvement in the detection accuracy of large targets, and there is room for further improvement in the optimization of scanning methods and the lightweight recognition speed. Future work can explore the combination of this method with large core convolution, wavelet transform, and other technologies, and at the same time design dynamically adjustable scanning paths according to the target size or local image complexity so as to enhance the generalization of targets of different sizes in the process of remote sensing detection. At the same time, it can be explored that the method can be used to integrate multi-modal features (such as infrared and visible light images) and noise suppression technology to improve model performance under more extreme scenarios with low signal-to-noise ratio.

## Figures and Tables

**Table 1:** Summary of datasets.

Dataset Name	Object Quantity	Object Distribution	Object Size	Category Quantity
DOTA-v1.0	263,427	3 per image	20 × 20–300 × 300	15
VEDAI	3575	3 per image	12 × 12–24 × 24	9
USOD	50,298	14.5 per image	8 × 8–16 × 16	1

**Table 2:** Effects of branch design strategies on each dataset: Paras, FLOPs, and mAP50.

Dataset	Branch Design	Paras	FLOPs	mAP50
USOD	C/4*4	17.15 M	73	89.60
	C/8*8	16.88 M	68	90.35
	C/4*8/2	19.29 M	80	89.98
	C/16*8*2	18.34 M	76	89.77
DOTAv1.0	C/4*4	19.01 M	81	70.07
	C/8*8	18.59 M	79	70.91
	C/4*8/2	20.97 M	89	69.98
	C/16*8*2	20.14 M	84	70.35
VEDAI	C/4*4	18.83 M	79	75.65
	C/8*8	17.94 M	77	77.94
	C/4*8/2	19.50 M	85	75.49
	C/16*8*2	18.99 M	81	76.33

**Table 3:** Effects of isolating the Gaussian mask alone on each dataset: Paras, FLOPs, and mAP50.

Dataset	Gaussian Mask	Paras	FLOPs	mAP50
USOD	configure	16.88 M	68	90.35
	unconfigure	16.81 M	67	89.82
DOTAv1.0	configure	18.59 M	79	70.91
	unconfigure	18.53 M	78	70.15
VEDAI	configure	17.94 M	77	77.94
	unconfigure	17.90 M	76	76.88

**Table 4:** Effects of different convolutional kernel sizes on each dataset: Paras, FLOPs, and mAP50.

Dataset	Kernel Size (1 × k, k × 1)	Paras	FLOPs	mAP50
USOD	3	16.88 M	68	90.35
	5	17.52 M	70	89.45
	7	20.15 M	75	89.98
	11	26.44 M	83	87.62
DOTAv1.0	3	18.59 M	79	70.91
	5	20.68 M	83	69.89
	7	25.42 M	88	70.32
	11	30.07 M	96	69.40
VEDAI	3	17.94 M	77	77.94
	5	19.33 M	80	76.85
	7	23.82 M	85	77.00
	11	27.37 M	91	76.13

**Table 5:** Impact of different component integration on the USOD dataset mAP50.

Structure	mAP50	Speed (ms)	Memory (MB)	LDblock	SS2D	MLP
(a)	88.87	3.5	1257	×	×	×
(b)	89.59	3.6	1289	√	×	×
(c)	89.83	3.9	1365	×	√	√
(d)	90.35	4.2	1418	√	√	√

**Table 6:** Comparison of results on DOTA-v1.0. The results highlighted in red and blue represent the best and second-best performance in each column, respectively.

Method	PL	BD	BR	GTF	SV	LV	SH	TC	BC	ST	SBF	RA	HA	SP	HC	mAP50	mAP50:95
DSSD	75.12	71.00	43.11	61.11	60.37	64.17	73.70	76.29	72.20	71.84	52.39	49.16	63.26	59.86	50.30	62.93	45.32
RefineDet	75.21	68.27	41.10	59.69	65.17	69.79	73.83	76.56	72.27	70.63	49.37	56.06	60.01	57.62	51.14	63.11	46.54
YOLOv3	67.67	64.25	36.68	65.72	59.99	64.17	69.45	70.28	65.68	66.91	48.67	50.02	57.98	60.35	58.61	60.43	44.21
YOLOv5	80.13	77.12	48.74	66.69	65.94	69.74	79.26	81.90	77.85	77.44	57.97	54.83	68.83	65.48	55.81	68.52	47.58
Gold-YOLO	80.54	73.25	45.91	61.99	70.01	74.10	79.39	82.07	78.65	75.86	55.06	58.84	66.09	59.92	43.42	67.01	47.45
BGF-YOLO	72.67	66.53	37.83	57.87	61.98	67.86	71.91	74.78	71.65	69.62	45.62	44.18	59.84	51.93	47.15	68.17	47.81
YOLOv8	76.05	72.66	44.96	74.14	68.19	72.50	77.76	78.53	73.93	75.24	56.90	58.24	66.26	68.65	66.92	68.73	47.99
FFCA-YOLO	80.43	78.37	52.75	65.41	73.43	77.94	78.53	81.49	78.00	80.17	60.20	57.32	62.21	73.16	67.33	70.12	49.88
DINO	79.70	75.46	47.67	65.62	64.85	68.67	78.19	80.82	76.81	76.34	56.88	53.76	67.76	64.41	55.04	67.47	47.69
DNTR	80.21	76.83	50.07	65.51	70.55	73.21	81.49	80.28	78.39	77.11	53.56	62.22	69.28	63.37	55.61	69.18	48.17
RFLA	73.98	71.65	48.08	67.44	67.09	71.79	74.69	77.15	72.73	73.55	54.28	56.16	69.42	67.61	64.29	67.39	47.32
YOLOv11	78.38	73.36	51.17	71.46	69.06	74.94	77.62	79.77	76.26	77.25	61.75	60.82	67.83	63.82	64.14	69.84	48.57
YOLOv12	75.94	75.08	53.42	65.99	70.21	72.92	72.10	77.33	73.28	69.91	60.86	58.62	66.10	68.29	66.33	68.43	47.78
YOLOv13	72.11	76.13	51.87	73.86	69.67	75.28	76.88	80.29	75.07	77.31	60.18	61.09	66.38	70.25	64.42	70.05	49.63
Mamba-YOLO	76.98	74.51	51.02	70.44	70.05	74.64	77.53	80.10	75.67	76.51	57.27	59.16	73.36	70.53	67.24	70.33	49.76
FBRT-YOLO	80.69	77.55	53.11	74.69	70.23	76.94	79.42	79.27	78.29	61.07	63.37	69.40	68.26	59.35	67.01	70.58	49.93
GMG-LDef Mamba-YOLO	81.54	78.37	53.91	75.47	70.81	76.83	79.79	82.25	80.60	79.28	54.77	55.93	69.94	66.70	57.47	70.91	50.39

**Table 7:** Comparison of results on VEDAI. The results highlighted in red and blue represent the best and second-best performance in each column, respectively.

Method	mAP50 (%)	Parameters (M)	FLOPs
YOLOv5	71.93	23.75	117
Gold-YOLO	75.02	28.76	122
BGF-YOLO	76.47	32.53	145
YOLOv8	74.39	28.14	125
FFCA-YOLO	75.18	11.20	82
YOLOv11	75.44	15.98	84
YOLOv12	74.39	20.46	108
YOLOv13	75.17	16.98	93
Mamba-YOLO [21]	76.98	41.81	151
FBRT-YOLO [56]	77.52	19.33	88
GMG-LDefmamba-YOLO	77.94	17.94	77

**Table 8:** Comparison of results on USOD. The results highlighted in red and blue represent the best and second-best performance in each column, respectively.

Method	Precision	Recall	mAP50	Speed (ms)	Memory (MB)
YOLOv5	85.80	79.41	83.95	5.6	1861
Gold-YOLO	88.77	82.10	87.95	5.2	1589
BGF-YOLO	88.18	82.56	88.24	5.3	1751
YOLOv8	87.65	82.01	87.40	4.8	1668
FFCA-YOLO	90.04	83.98	89.80	4.6	1655
YOLOv11	89.50	83.25	89.06	4.3	1446
YOLOv12	88.77	82.01	88.52	4. 7	1714
YOLOv13	89.38	82.53	88.89	4.5	1533
Mamba-YOLO	90.09	83.96	89.60	4.6	1729
FBRT-YOLO	90.33	83.95	89.83	4.4	1509
GMG-LDefmamba-YOLO	90.35	84.03	90.28	4.2	1418

**Table 9:** mAP50 breakdown by object scale on datasets. The results highlighted in red and blue represent the best and second-best performance in each column, respectively.

Model	Small Targets (mAP50, %)	Medium Targets (mAP50, %)	Large Targets (mAP50, %)	Extra-Large Targets (mAP50, %)
YOLOv11	61.29	77.08	88.36	93.01
Mamba-YOLO	63.17	75.33	88.67	94.13
YOLOv12	58.23	74.15	87.94	93.42
YOLOv13	59.05	73.82	89.51	92.76
GMG-LDefmamba-YOLO	72.58	81.34	91.29	95.05

## Data Availability

The original contributions presented in the study are included in the article; further inquiries can be directed to the corresponding author.
